# Evaluation of Dietary Probiotic (*Lactobacillus plantarum* BG0001) Supplementation on the Growth Performance, Nutrient Digestibility, Blood Profile, Fecal Gas Emission, and Fecal Microbiota in Weaning Pigs

**DOI:** 10.3390/ani11082232

**Published:** 2021-07-29

**Authors:** Huan Wang, In-Ho Kim

**Affiliations:** 1 Department of Animal Resource & Science, Dankook University, Cheonan 31116, Korea; wanghuan220203@163.com; 2 School of Biology and Food Engineering, Chuzhou University, Chuzhou 239012, China

**Keywords:** supplements, *Escherichia coli*, serum parameters, swine

## Abstract

**Simple Summary:**

Since antibiotics are banned in animal feed in many countries, probiotics have received more attention as reliable alternatives. We mainly study the effect of adding *Lactobacillus plantarum* BG0001 on the performance of weaned piglets for 42 days. The results: weaning pigs fed diet supplementation with *L. plantarum* BG0001 significantly improved the growth performance and fecal microbiota, and achieved similar effects as antibiotic growth promoters. Therefore, we consider that *L. plantarum* BG0001 will have a good role in replacing antibiotic growth promoters in swine feed. It can bring potentially huge economic income to the animal husbandry industry.

**Abstract:**

A total of 180, 4-week-old crossbred weaning piglets ((Yorkshire × Landrace) × Duroc; 6.67 ± 1.40 kg) were used in a 42 day experiment to evaluate the effect of dietary probiotics (*Lactobacillus plantarum* BG0001) on growth performance, nutrient digestibility, blood profile, fecal microbiota, and noxious gas emission. All pigs were randomly allotted to one of four treatment diets in a completely randomized block design. Each treatment had nine replicates with five pigs/pen (mixed sex) Designated dietary treatments were as: (1) basal diet (NC), (2) NC + 0.2% antibiotics (chlortetracycline) (PC), (3) NC + 0.1% *L. plantarum* BG0001 (*Lactobacillus plantarum* BG0001) (NC1), (4) NC + 0.2% *L. plantarum* BG0001 (NC2). On d 42, BW and G:F were lower (*p* < 0.05) in pigs fed NC diet compared with PC diet and probiotic diets. Throughout this experiment, the average daily gain increased (*p* < 0.05) in pigs when fed with PC and probiotic diets than the NC diet. The average daily feed intake was higher (*p* < 0.05) in pigs fed PC diet during day 0–7 and 22–42, and probiotic diets during day 0–7 compared with NC diet, respectively. The *Lactobacillus* count was increased and *Escherichia coli* count was decreased (*p* < 0.05) in the fecal microbiota of pigs fed probiotic diets, and *E. coli* were decreased (*p* < 0.05) when fed a PC diet compared with the NC diet on day 21. Moreover, the apparent total tract nutrient digestibility, blood profile, and the concentration of noxious gas emission had no negative effects by the probiotic treatments. In conclusion, dietary supplementation with *L. plantarum* BG0001 significantly improved the growth performance, increased fecal *Lactobacillus*, and decreased *E. coli* counts in weaning pigs.

## 1. Introduction

In the swine industry, weaning is one of the critical stages in piglets’ life. Weaning piglets may face several stressors during the transition, such as adaptation to a new environment, co-mingling with new litter mates, and feed switch from liquid to solid, which usually results in anorexia, diarrhea, and malnutrition and may be accompanied by severe transient growth restrictions [1,2]. These stresses may directly affect the economic benefits and productivity if not dealt with promptly. During the past last few decades, in order to overcome the series of problems, post-weaning piglet feed was usually supplemented with antibiotic additives as growth promoters [3]. However, in recent years many European countries have partially or completely banned the use of antibiotics in animal feed (especially to promote growth) due to the overuse of AGP, leading to the development of antibiotic resistance in the animal microbiota and the possibility to transfer antibiotic resistance genes from animal microbiota to human microbiota [4]. In South Korea, the use of antibiotics as growth promoters in animal feed has also been forbidden since 2011 [5]. Therefore, the safe and economical feed additives that can replace antibiotics have received more and more research and attention. In the current reports, the substitution of probiotics [6], prebiotics [7], enzymes [8], and organic acids [9] has received more attention due to their antagonism to a variety of microorganisms and significant growth promotion.

Among those substitutions, probiotics are considered to be very promising and important antibiotic growth-promoting alternative additives for weaned piglets [10]. Probiotics are defined as living microorganisms, which, consist of live cultures of bacillus, lactic acid bacteria (LAB), or yeast [11] and when applied in adequate amounts, confer health benefits for the host [12]. Studies have shown that the beneficial effects of probiotics on the host include increasing growth rate [13], improved digestibility [14], improved and prevention of diarrhea [6], modulatory effects on the immune system [15], and reduced harmful gas emissions [16], etc., At present, *Lactobacillus* has become a common probiotic supplementation in livestock feed [17]. *L. plantarum* is a facultative hetero-fermentative LAB, which usually ferments carbohydrates through the phosphoketolase pathway [18]. Different strains of *L. plantarum* have shown strong acid resistance and bile resistance [19] and can be found in a variety of environmental niches such as dairy, vegetables, and mammal gastrointestinal tracts, as well as being used in human food, aquaculture, and livestock feed as probiotics [20,21,22]. A previous study also indicates that *L. plantarum* has a beneficial effect on pigs [23,24,25,26].

Though previous studies used *L. plantarum* as a potential alternative to antibiotic growth promoter, to the best of our knowledge, the effect of *L. plantarum* strain, and *L. plantarum* on weaning pigs is still limited. In this study, the selected strain was the BG0001 strain isolated from traditional fermented foods (kimchi, salted fish and soy sauce, etc.), and used 16S rDNA to identify it as *L. plantarum* [27]. We hypothesize that this probiotic strain can improve the health and productivity of piglets by regulating the intestinal flora and stimulating the immune system. Therefore, the objective of this study was to evaluate the effects of dietary *L. plantarum* BG0001 on growth performance, nutrient digestibility, blood profile, fecal microbiota, and noxious gas emission in weaning pigs.

## 2. Materials and Methods

The experimental protocols describing the management and care of animals were reviewed and approved by the Animal Care and Use Committee of Dankook University, Cheonan, South Korea (DK-1-1921).

### 2.1. Source of Probiotic

The probiotics used in this study were obtained from a commercial company (Biogenoci Co., Ltd., Suwon, Korea) as *L. plantarum* BG0001. According to the supplier’s information, this product contained a spray-dried mixture of live *L. plantarum* of at least 1.30 × 10^7^ CFU/g of product in the form of powder. Probiotics were added before each feeding by thorough mixing with the feed.

### 2.2. Experimental Design, Animals, and Diets

A total of 180 4-week-old crossbred weaning pigs ((Yorkshire × Landrace) × Duroc; 6.67 ± 1.40 kg) were used in a 42-day experiment, and pigs received no creep feed before weaning. All pigs were randomly allotted to one of four treatment diets in a completely randomized block design. There were nine replicate pens per treatment with five pigs per pen (mixed sex). The dietary treatment was divided into three phases: Phase Ⅰ (days 0–7, corn–soybean meal basal diet providing 16.7472 MJ/kg and 20% crude protein), Phase Ⅱ (days 8–21, corn–soybean meal basal diet providing 16.32852 MJ/kg and 19% crude protein), and Phase Ⅲ (days 22–42, corn–soybean meal basal diet providing 15.90984 MJ/kg and 18.5% crude protein). The four treatments were: (1) basal diet (NC), (2) NC + 0.2% antibiotics (chlortetracycline) (PC), (3) NC + 0.1% *L. plantarum* BG0001 (*Lactobacillus plantarum* BG0001) (NC1), and (4) NC + 0.2% *L. plantarum* BG0001 (NC2). All nutrient in diets were formulated to meet or exceed the minimum requirements that was recommended by the National Research Council [28] (Table 1). All piglets were kept in an environmentally controlled room with a mechanical ventilation system. All test animals were fed the same basal diet during the experiment. At first, the temperature was maintained at 30 °C, thereafter it was reduced by 1 °C each week. Throughout the experiment, each pen was provided with a stainless-steel feeder and a nipple waterer to allow pigs ad libitum access to feed and water.

### 2.3. Sampling and Measurements

Individual pigs were weighed on d 0, 7, 21, and 42. Average daily gain (ADG), average daily feed intake (ADFI), and gain/feed (G:F) ratio were recorded.

To determine the apparent total tract digestibility (ATTD), chromium oxide (2 g/kg) was added to the diets as an indigestible marker for 7 days prior to fecal collection (d 15 and 36). On d 21 and 42, fecal samples were randomly collected from two pigs (1 gilt and 1 barrow; 18 pigs per treatment) per pen with body weights similar to the group’s average, and fecal samples were pooled by pen aim to obtain a representative composite sample. Collected fecal and feed samples were kept at −20 °C until analysis. All samples were thawed and dried (72 h; 57 °C) before the chemical analysis after they were ground to pass through a 1 mm screen. Dry matter (DM), nitrogen (N), and gross energy (GE) in the feed and feces were analyzed according to the procedures outlined by the AOAC [29]. Chromium and N were analyzed and determined via UV absorption spectrophotometry (Shimadzu, UV-1201, Kyoto, Japan) and Kjeltec 2300 Analyzer (Foss Tecator AB, Hoeganaes, Sweden) according to the methods from Hahn et al. [30]. The GE was determined by measuring the heat of combustion in the samples using a Parr 6100 oxygen bomb calorimeter (Parr Instrument Co., Moline, IL, USA). The ATTD was then calculated using the following formula:

(1) $$\mathrm{ATTD}\;\left(\%\right)=\left(1-\frac{\mathrm{Nf}\times \mathrm{Cd}}{\mathrm{Nd}\times \mathrm{Cf}}\right)\times 100\;$$

where Nf is the nutrient concentration in the feces (% DM), Nd is the nutrient concentration in the diet (% DM), Cd is the chromium concentration in the diet (% DM), and Cf is the chromium concentration in the feces (% DM).

On the same day (d 21 and 42), fresh fecal samples were collected directly by massaging the rectum of 2 pigs (1 gilt and 1 barrow) from each pen. Immediately it was kept on the ice pack and transported to the laboratory for microbiota analysis. A one gram fecal sample was diluted with 9 mL of 1% peptone broth (Becton, Dickinson) and was homogenized. *Escherichia coli* (*E. coli*) and *Lactobacillus* were enumerated by plating 10-fold serial dilution (in 1% peptone solution) onto MacConkey agar plates (Difco Laboratories, Detroit, MI, USA) and lactobacilli medium III agar plates (Medium 638; DSMZ, Braunschweig, Germany). To isolate the *E. coli* and *Lactobacillus*, it was anaerobically incubated at 39 °C for 48 h and 37 °C for 24 h before counting, respectively [31]. All bacteria were counted and log^10^ transformed before statistical analysis.

On d 21 and 42, the fresh fecal of at least 2 pigs from each pen by rectal massage was collected according to the method described by Cho et al. [32]. Fresh feces (300 g) were kept in 2.6 L plastic boxes and fermented for 24 h at 35 °C. Ammonia (NH_3_), hydrogen sulfide (H_2_S), total mercaptan (R.SH), and acetic acid (AC) in the fermented samples were measured by using a complex gas meter (MultiRAE Lite model PGM-6208, RAE System, Inc., Copenhagen, Denmark).

On day 21 and at the end of the experiment, two pigs (1 gilt and 1 barrow) were randomly selected from each pen (*n* = 36) per treatment, and 5 mL blood samples were collected via jugular venipuncture and stored into K_3_EDTA Vacuum tubes (Becton Dickinson Vacutainer Systems, Franklin Lakes, NJ, USA). After collection, blood samples were centrifuged (3000× *g*) for 15 min at 4 °C for the separation of serum. The WBC, RBC, and lymphocytes in the blood were analyzed using an analyzer (HITACHI 747, Tokyo, Japan).

### 2.4. Statistical Analysis

All data were analyzed as a randomized block design using the contrast model procedure of SAS (SAS Inst. Inc., Cary, NC, USA). Pen was used as an experimental unit. Contrasts used to separate treatment means were: NC vs. PC, PC vs. NC1, 2; and NC vs. NC1, 2. A probability level of *p* < 0.05 was statistically significant.

## 3. Results

### 3.1. Growth Performance

The results of the growth performance of weaning pigs are shown in Table 2. On day 42, PC group piglets showed higher (*p* < 0.05) BW compared to NC group piglets. Dietary *L. plantarum BG0001* supplement significantly (*p* < 0.05) increased the BW compared to those fed an NC diet. During day 7, the ADG and ADFI were significantly (*p* < 0.05) increased in pigs fed a PC diet than NC, NC1, and NC2, whereas NC diet pigs fed a graded level of *L. plantarum BG0001* had significantly improved (*p* < 0.05) the ADG and ADFI. On day 21 and 42, pigs fed a PC diet had a trend (*p* < 0.05) to significantly increase (*p* < 0.05) ADG and ADFI compared to the NC group, respectively, whereas compared to the NC diet pigs increased levels of *L. plantarum* had a trend to significantly increase (*p* < 0.05) the ADG. During the overall experimental period, PC group piglets had higher ADG and G:F compared to those fed NC diet. When comparing to the NC group, NC1 and 2 group piglets showed increased (*p* < 0.05) ADG, and G:F.

### 3.2. Nutrient Digestibility and Blood Profile

The results of the apparent total tract nutrient digestibility (DM, N, and GE) and blood profile (WBC, RBC, Lymphocyte) of weaning pigs are shown in Table 3 and Table 4. There were no significant effects (*p* > 0.05) observed among the treatments.

### 3.3. Fecal Microbiota and Noxious Gas Emission

The results of the fecal *Lactobacillus* and *E. coli* count, and noxious gas emission (NH_3_, H_2_S, R.SH, and AC) of weaning pigs are shown in Table 5. The fecal *Lactobacillus* counts was significantly increased (*p* < 0.05) in pigs fed NC1 and 2 compared to those fed NC diet. Moreover, compared to NC group pigs receive *L. plantarum* BG0001 supplement showed reduced (*p* < 0.05) *E. coli* count at day 21. However, there were no difference (*p* > 0.05) observed on fecal noxious gas emission throughout the trial.

## 4. Discussion

The weaning process may limit piglet feed intake, absorption, and digestibility leading to gut health problems. In the context of the ban on the sub-therapeutic use of antibiotics, previous studies have shown that probiotic LAB as the most commonly used supplement that could contribute to reducing weaning stress [33]. In the current experiment, supplementing *L. plantarum* BG0001 significantly improved the growth performance of piglets, and the results showed that the effect of probiotics was the same as that of antibiotic growth promoter except for the first week of the experiment. In agreement with the current findings, Suo et al. [26] reported that *L. plantarum* ZJ316 additive (1 × 10^9^ CFU/day) isolated from infant fecal samples has significantly improved the growth performance of weaned piglets. Cai et al.’s [34] study results showed a beneficial effect of *L. plantarum* (5 × 10^11^ CFU kg^−1^) diet supplementation on growth performance in weaned piglets. In addition, Thu et al.’s [2] research also showed that the metabolites combination of *L. plantarum* strains could be used as a growth promoter, which are a potential alternative to antibiotics. More, studies have reported the beneficial effects of *L. plantarum* on the human body. For example, Kaushik et al. [35] found that *L. plantarum* (Lp9) could survive the harsh acidic pH prevalent in an adult human stomach and showed health-promoting properties. This beneficial effect on growth performance has also been found in post-weaning rats [36] and broiler chickens [33], however, the reports were inconsistent. Jones et al. [37] reported that feeding increasing dietary levels of *L. plantarum* had no impact on nursery pig performance. The inconsistent result may be related to the animal species, health status of the pigs, nutritional level, and activities of *L. plantarum*. Generally, the improvement of nutrient digestibility is a reason for the improved performance of weaned piglets. However, we found that nutrient digestibility did not show a significant difference from dietary supplements with probiotics in the finishing pig. similarly, O’Shea et al. [38] also found that *L. plantarum* supplemented in diets did not show a significant difference in nutrient digestibility.

The results of this study showed no effects on blood profile, including WBC, RBC, and Lymphocyte among the treatment groups, which is in agreement with previous studies of Liu et al. [39] and Wang et al. [40]. Moreover, Tufarelli et al. [41] reported that supplementation, which included *L. plantarum* DSM probiotic complex, had no effect on RBC, WBC, Lymphocyte in pig blood. However, there are few research reports on the mechanism and the effect of *L. plantarum* on the blood profile.

Probiotics maintain the balance of the intestinal ecosystem by promoting beneficial bacteria and inhibiting harmful bacteria and are closely related to health conditions [42]. Previous studies have shown that *L. plantarum* (3 × 10^10^) can greatly reduce the incidence of diarrhea in weaned piglets [43]. In the current study, we found that the supplementation of *L. plantarum* BG0001 in the diet of weaning pigs has significantly increased the number of *Lactobacillus* and reduced *E. coli* on day 21. Similarly, Pupa et al. [44] reported that *L. plantarum* (22F, 25F) and *Pediococcus acidilactici* (72N) supplementation in pigs significantly increased viable lactobacilli and reduced enterobacterial counts. Betancur et al. [45] found that supplementation of the *L. plantarum CAM6* to sows decreased the diarrhea incidence of offspring. Moreover, supplementation with *Lactobacillus plantarum*-based combination additives has the most obvious inhibitory effect on *E. coli* O8:K88 cells that adhere to the jejunum and colon mucosa in piglet was found by Nemcová et al. [46]. However, different findings have been reported in the literature. Jones et al. [37] reported that feeding increasing dietary levels of *L. plantarum* had no evidence of an impact on nursery pig performance. In contrast, Wang et al. [47] reported that *L. plantarum* ZLP001 fortifies the intestinal barrier in weaning pigs by enhancing epithelial defense functions and modulating gut flora. *Lactobacillus* spp. enhances the immune system by promoting the expression of anti-inflammatory cytokines or inhibiting the expression pro-inflammatory cytokines. Some reports have shown that phytomycin produced by *L. plantarum* has a broader antimicrobial spectrum of inhibition, such as plantaricin 163 can inhibit some Gram-positive bacteria and some Gram-negative bacteria, and plantaricin LP84 can inhibit both Gram-positive bacteria and Gram-negative bacteria as well as the production of food-borne pathogenic and spoilage bacteria [48,49].

Fecal noxious gas emission has become one of the major air pollutions in modern concentrative pig production [16]. Excessive harmful gas emissions will cause disruptions in the ecological balance [50]. We found that dietary supplementation with *L. plantarum* did not affect harmful gas emission in feces. Furthermore, probiotic supplementation in the diet marginally decreased H_2_S (*p* = 0.062) compared with the basal diet on day 21, but was not significantly different. In addition, fecal noxious gas emission is associated with nutrient digestibility because the higher digestibility may result in a lower substrate for the microbial fermentation in the large intestine, which consequently decreases fecal noxious gas emission [51]. This verifies our research results, in which there was no significant difference in nutrient digestibility and noxious gas emission. It was also suggested that noxious gas emission is related to harmful intestinal bacteria population [50]. In the current study, non-significant effects on noxious gas emissions when fed diet supplemented either with antibiotics or *L. plantarum* may also indicate that the weaning piglets were in good physical condition.

## 5. Conclusions

The weaning pressure of piglets seriously affects the balance of intestinal flora and the healthy growth of the body. The results of this study indicated that weaning pigs fed diet supplemented with *L. plantarum* BG0001 had significantly improved the growth performance and fecal microbiota, and have achieved similar effects as antibiotic growth promoters. On the other hand, the results of this study also showed that the addition of antibiotics and probiotics (*L. plantarum* BG0001) in the diet of weaning pigs had no negative effect on nutrient digestibility (DM, N, and GE), blood profile (WBC, RBC, Lymphocyte), and noxious gas emission. Therefore, we considered that *L. plantarum* BG0001 will have a good role in replacing antibiotic growth promoters in swine feed. Moreover, it is foreseeable that the synergistic probiotic complex based on this experimental strain may have a higher effect and economic return in the application of alternative antibiotics.

## Figures and Tables

**Table 1:** Composition of basal weaning pig diets (as fed-basis).

Items	Phase 1	Phase 2	Phase 3
Ingredients, %			
Extruded corn	37.29	47.80	55.83
Soybean meal (dehulled)	12.00	18.00	24.00
Fermented soybean meal	10.00	8.00	5.00
LT-fish meal	7.60	2.70	-
Soy oil	3.13	3.20	3.25
Dicalcium phosphate	1.24	1.34	1.63
Limestone	0.60	0.74	0.82
Sugar	3.00	2.00	2.00
Whey protein	11.00	8.00	3.00
Lactose	12.80	6.70	3.00
L-lysine–HCL	0.35	0.46	0.48
DL-methionine	0.18	0.17	0.19
Threonine	0.21	0.29	0.20
Choline chloride 50%	0.10	0.10	0.10
Salt	0.10	0.10	0.10
Premix ^1^	0.40	0.40	0.40
Nutrients, % ^2^			
Crude protein	20.0	19.0	18.5
Crude fat	5.40	4.80	4.20
Calcium	0.80	0.75	0.75
Phosphorus	0.70	0.65	0.65
Digestible energy, kcal/kg	4000	3900	3800
Lysine	1.60	1.50	1.40
Methionine	0.48	0.45	0.42
Lactose	20.0	12.0	5.0

^1^ Premix provided the following per kilograms of diet: Fe, 100 mg as ferrous sulfate; Cu, 17 mg as copper sulfate; Mn, 17 mg as manganese oxide; I, 0.5 mg as potassium iodide; and Se, 0.3 mg as sodium selenite; vitamin A, 10,800 IU; vitamin D3, 4000 IU; vitamin E, 40 IU; vitamin K3, 4 mg; vitamin B1, 6 mg; vitamin B2, 12 mg; vitamin B6, 6 mg; vitamin B12, 0.05 mg; biotin, 0.2 mg; folic acid, 2 mg; niacin, 50 mg; D-calcium pantothenate, 25 mg. ^2^ Nutrients levels were calculated values.

**Table 2:** Effect of dietary *Lactobacillus plantarum* BG0001 supplementation on growth performance in weaning pigs ^1^.

Items	NC	PC	NC1	NC2	SEM ^2^	*p*-Value		
						NC vs. PC	PC vs. NC1, 2	NC vs. NC1, 2
BW, kg								
Initial	6.67	6.67	6.67	6.66	0.002	0.623	0.259	0.131
D 42	23.39	24.65	24.19	24.27	0.40	0.002	0.181	0.017
D 0–7								
ADG, g	217	246	228	230	3.69	<0.001	0.001	0.011
ADFI, g	244	272	252	257	3.75	<0.001	0.001	0.033
G:F	0.891	0.904	0.905	0.895	0.007	0.165	0.872	0.150
D 8–21								
ADG, g	377	394	391	392	4.43	0.007	0.654	0.008
ADFI, g	485	500	492	494	9.54	0.272	0.571	0.480
G:F	0.780	0.788	0.798	0.795	0.008	0.233	0.670	0.075
D 22–42								
ADG, g	472	511	494	500	10.43	0.011	0.278	0.051
ADFI, g	713	758	735	741	14.81	0.038	0.283	0.176
G:F	0.662	0.674	0.673	0.675	0.005	0.080	0.926	0.054
D 0–42								
ADG, g	478	514	499	503	7.65	0.002	0.183	0.016
ADFI, g	559	586	573	576	9.63	0.053	0.325	0.201
G:F	0.855	0.876	0.871	0.873	0.004	0.001	0.489	0.002

^1^ Abbreviation: NC, basal diet; PC, NC + 0.2% antibiotic; NC1, NC + 0.1% *L. plantarum* BG0001; NC2, NC + 0.2% *L. plantarum* BG0001. ^2^ SEM: pooled standard error of the means.

**Table 3:** Effect of dietary *Lactobacillus plantarum* BG0001 supplementation on nutrient digestibility in weaning pigs ^1^.

Items, %	NC	PC	NC1	NC2	SEM ^2^	*p*-Value		
						NC vs. PC	PC vs. NC1, 2	NC vs. NC1, 2
D 21								
DM	83.72	84.55	83.85	83.99	0.58	0.319	0.383	0.777
N	81.68	82.50	81.82	81.89	0.56	0.315	0.359	0.804
GE	82.81	83.42	82.76	82.91	0.58	0.470	0.422	0.976
D 42								
DM	80.20	82.05	81.07	81.43	0.81	0.123	0.429	0.305
N	79.25	80.53	79.98	80.47	0.68	0.197	0.712	0.257
GE	80.08	81.07	80.64	80.84	0.76	0.369	0.724	0.491

^1^ Abbreviation: NC, basal diet; PC, NC + 0.2% antibiotic; NC1, NC + 0.1% *L. plantarum* BG0001; NC2, NC + 0.2% *L. plantarum* BG0001. ^2^ SEM: pooled standard error of the means.

**Table 4:** Effect of dietary Lactobacillus plantarum BG0001 supplementation on blood profile in weaning pigs ^1^.

Items, %	NC	PC	NC1	NC2	SEM ^2^	*p*-Value		
						NC vs. PC	PC vs. NC1, 2	NC vs. NC1, 2
D 21								
WBC, 10^3^/μL	18.31	16.04	17.94	17.01	0.86	0.094	0.204	0.449
RBC, 10^6^/μL	5.88	5.70	6.10	6.22	0.46	0.779	0.430	0.634
Lymphocyte, %	50.00	50.50	51.60	53.40	4.25	0.939	0.701	0.638
D 42								
WBC, 10^3^/μL	19.11	19.36	20.09	19.63	0.78	0.123	0.429	0.305
RBC, 10^6^/μL	5.89	6.10	6.09	6.05	0.37	0.197	0.712	0.257
Lymphocyte, %	54.40	52.80	56.80	56.50	1.70	0.369	0.724	0.491

^1^ Abbreviation: NC, basal diet; PC, NC + 0.2% antibiotic; NC1, NC + 0.1% *L. plantarum* BG0001; NC2, NC + 0.2% *L. plantarum* BG0001. ^2^ SEM: pooled standard error of the means.

**Table 5:** Effect of dietary *Lactobacillus plantarum* BG0001 supplementation on fecal microbiota and fecal noxious gas emission in weaning pigs ^1^.

Items	NC	PC	NC1	NC2	SEM ^2^	*p*-Value		
						NC vs. PC	PC vs. NC1, 2	NC vs. NC1, 2
Fecal microbiota (log_10_ CFU/g)								
D 21								
*Lactobacillus*	6.91	6.92	6.99	6.98	0.03	0.788	0.087	0.045
*E. coli*	6.45	6.25	6.33	6.32	0.05	0.009	0.268	0.049
D 42								
*Lactobacillus*	7.16	7.13	7.22	7.20	0.06	0.747	0.283	0.479
*E. coli*	6.10	5.95	6.00	6.05	0.09	0.255	0.513	0.502
Fecal noxious gas emission								
D 21								
NH_3_	2.2	2.0	2.9	2.8	0.5	0.718	0.172	0.320
H_2_S	3.7	3.4	2.6	2.9	0.4	0.601	0.166	0.062
R.SH	2.0	1.3	2.0	1.7	0.4	0.272	0.332	0.752
AC	3.2	2.1	2.9	2.6	0.4	0.180	0.246	0.328
D 42								
NH_3_	2.9	3.3	3.6	3.6	1.1	0.797	0.834	0.614
H_2_S	5.4	4.1	4.2	4.7	0.5	0.107	0.584	0.168
R.SH	3.0	4.7	4.1	3.2	1.1	0.312	0.475	0.635
AC	3.5	3.4	3.3	3.0	0.2	0.748	0.464	0.281

^1^ Abbreviation: NC, basal diet; PC, NC + 0.2% antibiotic; NC1, NC + 0.1% *L. plantarum* BG0001; NC2, NC + 0.2% *L. plantarum* BG0001. ^2^ SEM: pooled standard error of the means.

## Data Availability

Not applicable.

## References

[1] Campbell J.M., Crenshaw J.D., Polo J. (2013). The biological stress of early weaned piglets. J. Anim. Sci. Biotechnol..

[2] Thu T.V., Loh T.C., Foo H.L., Yaakub H., Bejo M.H. (2011). Effects of liquid metabolite combinations produced by Lactobacillus plantarum on growth performance, faeces characteristics, intestinal morphology and diarrhoea incidence in postweaning piglets. Trop. Anim. Health Prod..

[3] Torrallardona D. (2009). Spray dried animal plasma as an alternative to antibiotics in weanling pigs-a review. Australas. J. Anim. Sci..

[4] Mingmongkolchai S., Panbangred W. (2018). Bacillus probiotics: An alternative to antibiotics for livestock production. J. Appl. Microbiol..

[5] Global Agricultural Information Network (2011). Global Agricultural Information Network: Korea Phases Out Antibiotic Usage in Compound Feed. http://gain.fas.usda.gov/Recent%20GAIN%20Publications/Korea%20Phases%20Out%20Antibiotic%20Usage%20in%20Compound%20Feed_Seoul_Korea%20-%20Republic%20of_7-13-2011.pdf.

[6] Sun Y., Duarte M.E., Kim S.W. (2021). Dietary inclusion of multispecies probiotics to reduce the severity of post-weaning diarrhea caused by Escherichia coli F18+ in pigs. Anim. Nutr..

[7] Radzikowski D., Milczarek A. (2021). Selected feed additives used in pig nutrition. J. Cent. Eur. Agric..

[8] Torres-Pitarch A., Manzanilla E.G., Gardiner G.E., O’Doherty J.V., Lawlor P.G. (2019). Systematic review and meta-analysis of the effect of feed enzymes on growth and nutrient digestibility in grow-finisher pigs: Effect of enzyme type and cereal source. Anim. Feed Sci. Technol..

[9] Wei X., Bottoms K.A., Stein H.H., Blavi L., Bradley C.L., Bergstrom J., Knapp J., Story R., Maxwell C., Tsai T. (2021). Dietary Organic Acids Modulate Gut Microbiota and Improve Growth Performance of Nursery Pigs. Microorganisms.

[10] Azad M., Kalam A., Sarker M., Li T., Yin J. (2018). Probiotic species in the modulation of gut microbiota: An overview. BioMed Res. Int..

[11] Stein H. Feeding the pigs’ immune system and alternatives to antibiotics. Proceedings of the London Swine Conference.

[12] FAO/WHO (2006). Probiotics in Food. Health and Nutritional Properties and Guidelines for Evaluation. FAO Food and Nutrition Paper 85.

[13] Wang X., Tsai T., Wei X., Zuo B., Davis E., Rehberger T., Hernandez S., Jochems E.J., Maxwell C.V., Zhao J. (2021). Effect of Lactylate and Bacillus subtilis on growth performance, peripheral blood cell profile, and gut microbiota of nursery pigs. Microorganisms.

[14] Hu J., Kim Y.H., Kim I.H. (2021). Effects of two Bacillus strains probiotic supplement on reproduction performance, nutrient digestibility, blood profile, fecal score, excreta odor contents and fecal microflora in lactation sows, and growth performance in sucking piglets. Livest. Sci..

[15] Zhaxi Y., Meng X., Wang W., Wang L., He Z., Zhang X., Pu W. (2020). Duan-nai-An, A Yeast probiotic, improves intestinal Mucosa integrity and immune function in Weaned piglets. Sci. Rep..

[16] Nguyen D.H., Nyachoti C.M., Kim I.H. (2019). Evaluation of effect of probiotics mixture supplementation on growth performance, nutrient digestibility, faecal bacterial enumeration, and noxious gas emission in weaning pigs. Ital. J. Anim. Sci..

[17] McCoy S., Gilliland S.E. (2007). Isolation and characterization of Lactobacillus species having potential for use as probiotic cultures for dogs. J. Food Sci..

[18] Da Silva Sabo S., Vitolo M., González J.M.D., de Souza Oliveira R.P. (2014). Overview of Lactobacillus plantarum as a promising bacteriocin producer among lactic acid bacteria. Food Res. Int..

[19] De Vries M.C., Vaughan E.E., Kleerebezem M., de Vos W.M. (2006). Lactobacillus plantarum—Survival, functional and potential probiotic properties in the human intestinal tract. Int. Dairy J..

[20] Gewaily M.S., Shukry M., Abdel-Kader M.F., Alkafafy M., Farrag F.A., Moustafa E.M., Van Doan H., Abd-Elghany M.F., Abdelhamid A.F., Eltanahy A. (2021). Dietary Lactobacillus plantarum Relieves Nile Tilapia (Oreochromis niloticus) Juvenile from Oxidative Stress, Immunosuppression, and Inflammation Induced by Deltamethrin and Aeromonas hydrophila. Front. Mar. Sci..

[21] Zhang Y., Liu W., Wei Z., Yin B., Man C., Jiang Y. (2021). Enhancement of functional characteristics of blueberry juice fermented by Lactobacillus plantarum. LWT.

[22] Lin X., Xia Y., Yang Y., Wang G., Zhou W., Ai L. (2020). Probiotic characteristics of Lactobacillus plantarum AR113 and its molecular mechanism of antioxidant. LWT.

[23] Geng T., Su S., Sun K., Zhao L., Zhao Y., Bao N., Pan L., Sun H. (2021). Effects of feeding a Lactobacillus plantarum JL01 diet on caecal bacteria and metabolites of weaned piglets. Lett. Appl. Microbiol..

[24] Betancur C., Martínez Y., Merino-Guzman R., Hernandez-Velasco X., Castillo R., Rodríguez R., Tellez-Isaias G. (2020). Evaluation of oral administration of Lactobacillus plantarum CAM6 strain as an alternative to antibiotics in weaned pigs. Animals.

[25] Cui K., Wang Q., Wang S., Diao Q., Zhang N. (2019). The facilitating effect of tartary buckwheat flavonoids and Lactobacillus plantarum on the growth performance, nutrient digestibility, antioxidant capacity, and fecal microbiota of weaned piglets. Animals.

[26] Suo C., Yin Y., Wang X., Lou X., Song D., Wang X., Gu Q. (2012). Effects of lactobacillus plantarumZJ316 on pig growth and pork quality. BMC Vet. Res..

[27] Park D.-M., Kang K.-G., Kwon D.-H., Park J.-B., Yang B.-K., Ha S.-J. Isolation of Lactobacillus plantarum BG0001 Producing Bacteriocin and Evaluation of Antimicrobial Activity. Korean Society of Biological Engineering Spring Conference and International Symposium.

[28] Hahn T.W., Lohakare J.D., Lee S.L., Moon W.K., Chae B.J. (2006). Effects of supplementation of β-glucans on growth performance, nutrient digestibility, and immunity in weanling pigs. J. Anim. Sci..

[29] NRC (2012). Nutrient Requirements of Swine.

[30] AOAC—Association of Official Analytical Chemists (2005). Official Methods of Analysis.

[31] Sampath V., Heon Baek D., Shanmugam S., Kim I.H. (2021). Dietary Inclusion of Blood Plasma with Yeast (Saccharomyces cerevisiae) Supplementation Enhanced the Growth Performance, Nutrient Digestibility, Lactobacillus Count, and Reduced Gas Emissions in Weaning Pigs. Animals.

[32] Thanh N.T., Loh T.C., Foo H.L., Hair-Bejo M., Azhar B.K. (2009). Effects of feeding metabolite combinations produced by Lactobacillus plantarum on growth performance, faecal microbial population, small intestine villus height and faecal volatile fatty acids in broilers. Br. Poult. Sci..

[33] Cai Y.H., Aguilar Y.M., Yu L., Wang Y., Liu H.B., Liu G., Yin Y.L. (2014). Effects of dietary supplementation of Lactobacillus plantarum on growth performance and serum concentration of amino acids in weaned piglets. Anim. Nutr. Feed Technol..

[34] Kaushik J.K., Kumar A., Duary R.K., Mohanty A.K., Grover S., Batish V.K. (2009). Functional and probiotic attributes of an indigenous isolate of Lactobacillus plantarum. PLoS ONE.

[35] Foo H.L., Loh T.C., Law F.L., Lim Y.Z., Kufli C.N., Rusul G. (2003). Effects of feeding Lactobacillus plantarum I-UL4 isolated from Malaysian Tempeh on growth performance, faecal flora and lactic acid bacteria and plasma cholesterol concentrations in postweaning rats. Food Sci. Biotechnol..

[36] Jones A.M., Woodworth J.C., DeRouchey J.M., Dritz S.S., Tokach M.D., Goodband R.D. (2017). Effect of feeding varying levels of Lactobacillus Plantarum on nursery pig performance. J. Anim. Sci..

[37] O’Shea C.J., Sweeney T., Bahar B., Ryan M.T., Thornton K., O’Doherty J.V. (2012). Indices of gastrointestinal fermentation and manure emissions of growing-finishing pigs as influenced through singular or combined consumption of Lactobacillus plantarum and inulin. J. Anim. Sci..

[38] Liu X., Kim S.H., Kim I.H. (2020). Effects of the combination of multistrain probiotics and Castanea crenata shell extract on growth performance, nutrient digestibility, fecal microbial shedding, meat quality, noxious gas emissions, and blood parameters in finishing pigs. Livest. Sci..

[39] Wang H., Kim K.P., Kim I.H. (2019). Influence of Bacillus subtilis GCB-13-001 on growth performance, nutrient digestibility, blood characteristics, faecal microbiota and faecal score in weanling pigs. J. Anim. Physiol. Anim. Nutr..

[40] Tufarelli V., Crovace A.M., Rossi G., Laudadio V. (2017). Effect of a dietary probiotic blend on performance, blood characteristics, meat quality and faecal microbial shedding in growing-finishing pigs. S. Afr. J. Anim. Sci..

[41] Ng S.C., Hart A.L., Kamm M.A., Stagg A.J., Knight S.C. (2009). Mechanisms of action of probiotics: Recent advances. Inflamm. Bowel Dis..

[42] Pieper R., Janczyk P., Urubschurov V., Hou Z., Korn U., Pieper B., Souffrant W.B. (2010). Effect of Lactobacillus plantarum on intestinal microbial community composition and response to enterotoxigenic *Escherichia coli* challenge in weaning piglets. Livest. Sci..

[43] Pupa P., Apiwatsiri P., Sirichokchatchawan W., Pirarat N., Maison T., Koontanatechanon A., Prapasarakul N. (2021). Use of Lactobacillus plantarum (strains 22F and 25F) and Pediococcus acidilactici (strain 72N) as replacements for antibiotic-growth promotants in pigs. Sci. Rep..

[44] Betancur C., Martínez Y., Tellez-Isaias G., Castillo R., Ding X. (2021). Effect of Oral Administration with Lactobacillus plantarum CAM6 Strain on Sows during Gestation-Lactation and the Derived Impact on Their Progeny Performance. Mediat. Inflamm..

[45] Nemcová R., Bomba A., Gancarčíková S., Reiffová K., Guba P., Koščová J., Jonecova Z., Scirankova L., Bugarský A. (2007). Effects of the administration of lactobacilli, maltodextrins and fructooligosaccharides upon the adhesion of *E. coli* O8: K88 to the intestinal mucosa and organic acid levels in the gut contents of piglets. Vet. Res. Commun..

[46] Wang J., Ji H., Wang S., Liu H., Zhang W., Zhang D., Wang Y. (2018). Probiotic Lactobacillus plantarum promotes intestinal barrier function by strengthening the epithelium and modulating gut microbiota. Front. Microbiol..

[47] Hu M., Zhao H., Zhang C., Yu J., Lu Z. (2013). Purification and characterization of plantaricin 163, a novel bacteriocin produced by Lactobacillus plantarum 163 isolated from traditional Chinese fermented vegetables. J. Agric. Food Chem..

[48] Suma K., Misra M.C., Varadaraj M.C. (1998). Plantaricin LP84, a broad spectrum heat-stable bacteriocin of Lactobacillus plantarum NCIM 2084 produced in a simple glucose broth medium. Int. J. Food Microbiol..

[49] Ferket P.R., Van Heugten E., Van Kempen T.A.T.G., Angel R. (2002). Nutritional strategies to reduce environmental emissions from nonruminants. J. Anim. Sci..

[50] Yan L., Meng Q.W., Kim I.H. (2011). The effect of an herb extract mixture on growth performance, nutrient digestibility, blood characteristics and fecal noxious gas content in growing pigs. Livest. Sci..

[51] Cho J.H., Chen Y.J., Min B.J., Yoo J.S., Wang Y., Kim I.H. (2008). Effects of reducing dietary crude protein on growth performance, odor gas emission from manure and blood urea nitrogen and IGF-1 concentrations of serum in nursery pigs. Anim. Sci. J..

